# Advancing access to genome sequencing for rare genetic disorders: recent progress and call to action

**DOI:** 10.1038/s41525-024-00410-2

**Published:** 2024-03-27

**Authors:** Vaidehi Jobanputra, Brock Schroeder, Heidi L. Rehm, Wei Shen, Elizabeth Spiteri, Ghunwa Nakouzi, Stacie Taylor, Christian R. Marshall, Linyan Meng, Stephen F. Kingsmore, Katarzyna Ellsworth, Euan Ashley, Ryan J. Taft, on behalf of the Medical Genome Initiative

**Affiliations:** 1 Molecular Diagnostics, New York Genome Center, New York, NY USA; 2 Pathology and Cell Biology, Columbia University Irving Medical Center, New York, NY USA; 3 Market Access, Illumina Inc., San Diego, CA USA; 4 Medical and Population Genetics, Broad Institute of MIT and Harvard, Cambridge, MA USA; 5 Center for Genomic Medicine, Massachusetts General Hospital, Boston, MA USA; 6 Department of Laboratory Medicine and Pathology, Mayo Clinic, Rochester, MN USA; 7 Clinical Genomics, Department of Pathology, Stanford Medicine, Palo Alto, CA USA; 8 HudsonAlpha Clinical Services Lab, LLC, HudsonAlpha Institute for Biotechnology, Birmingham, AL USA; 9 Medical Affairs, Illumina Inc., San Diego, CA USA; 10 Division of Genome Diagnostics, Pediatric Laboratory Medicine Department, The Hospital for Sick Children, Toronto, ON Canada; 11 Molecular and Human Genetics, Baylor College of Medicine, Houston, TX USA; 12 Rady Children’s Institute for Genomic Medicine, Rady Children’s Hospital, San Diego, CA USA; 13 Stanford Center for Undiagnosed Diseases, Stanford University, Stanford, CA USA; 14 Medical Genomics Research, Illumina Inc., San Diego, CA USA

**Keywords:** Genetic testing, Genetic testing, Genome, Health policy

## Introduction

Epidemiologic studies estimate that 2–6% of the global population is affected by a rare disease, up to 80% of which are genetic in origin^1,2^. Diagnostic delays can result in significant burdens including missed opportunities for intervention, unnecessary procedures and treatments, and an emotional toll on families and their care providers^3^.

Genome sequencing (GS) provides a comprehensive profile of genetic variants associated with disease, including assessment of single nucleotide variants (SNV), indels, copy-number and structural variants, repeat expansions, and mitochondrial genome variation. The diagnostic potential of GS is underscored by the increasing evidence that it can end the so-called diagnostic odyssey for up to ~20-60% of neonates and ~17-40% of pediatric patients with a suspected genetic disease^4^. GS testing often leads to measurable changes in management, with studies suggesting that up to 77% of patients receive a change in care as a result of receiving diagnostic genome findings^5–12^. Health economic studies examining the incremental net benefit of GS in comparison to other genetic tests indicate that first-line GS can be a cost-effective strategy in patients with suspected rare diseases^7,9^. These and other results have led the Medical Genome Initiative to argue that GS should be applied as a first-line test for patients with a suspected rare genetic disease, and have supported the inclusion of GS in clinical practice guidelines published by the American College of Medical Genetics and Genomics (ACMG) in 2021^13^ and the European Society for Human Genetics (ESHG) in 2022^14^. Until recently, however, there has been limited government and payer support of GS testing.

## Recent advancements in access

In the last three years there has been a pronounced increase in the number of national, regional, and commercial policies that endorse GS testing for individuals with a suspected genetic disease. Backed by evidence generated by the Genomics England 100,000 genomes program, NHS-England became the first large-scale single-payer system to support systematic utilization of GS for patients with a suspected genetic disease, including those with intellectual disability, neuromuscular disorders, and primary immunodeficiencies^15^. In Australia the health technology assessment body for devices and diagnostic tests, the Medical Services Advisory Committee, has recommended exome and genome sequencing for intellectual disability, congenital anomalies^16^, suspected mitochondrial disease^17^, and hearing impairment^18^. There have also been changes in coverage in Western Europe: Germany has commissioned genomic testing through their rare disease network (NAMSE) and will expand GS implementation into routine care in early 2024, and Switzerland and Norway have commissioned GS in their national fee schedules^19^. Coverage changes are anticipated in at least half a dozen additional countries, including France, Israel, Spain, the Nordics and Japan, which are engaged in coverage pilots or large-scale evidence generation efforts^20,21^.

The US, with a population exceeding 330 million, operates one of the world’s most complex healthcare systems^22^. Government-funded Medicare and Medicaid, which address elderly and low-income patients, respectively, cover ~45% of the population, with commercial insurance covering the remainder. Out-of-pocket expenses are not insignificant, however, and account for ~10% of total healthcare spending^23^.

Until recently, there was limited coverage for GS in the US. Commercial insurance policies with allowance for GS covered less than 3 M lives. In early 2023, however, UnitedHealthcare (UHC), the largest commercial health insurer in the US, implemented a policy that expanded coverage to ~27 M commercial lives and ~7 M Managed Medicaid lives^24^, enabling GS testing in the pediatric population across a wide range of possible genetic disease indications, including multiple congenital anomalies, intellectual disability, global developmental delay and early-onset epileptic encephalopathy, and of children with select constellations of less severe phenotypes. Several other payers, including Cigna^25^, Select Health, and Geisinger have also recently updated their coverage policies and are now covering GS for select patients with indications of a genetic disease. There are now Medicaid coverage policies for rapid diagnostic GS for hospitalized infants and children in nine US states with a childhood population of 24 M. With these changes, the total number of covered lives in the US now exceeds 50 M. A request in the Fiscal Year 2023 Omnibus Appropriations Bill that the Centers for Medicare and Medicaid Services (CMS) develop guidance for state health officials on best practices for incorporating GS and other genetic testing technologies into their Medicaid and Children’s Health Insurance Program (CHIP), and to investigate how such testing fits into the Early and Periodic Screening, Diagnostic and Treatment (EPSDT) benefit, may further improve both coverage and access for Medicaid patients.

## Remaining issues and call to action

Despite these advancements, only a small fraction of the population is covered for GS, and such testing is largely inaccessible in low- and middle-income countries. Indeed, even in geographies where genetic testing is well covered, there is substantial under-utilization^26^ often exacerbated by limited physician awareness, long wait times for specialist consultations, and patient and physician challenges navigating the health insurance system. To address these gaps, and to accelerate access to a precision diagnosis for all patients with a genetic disease, we recommend the following collective actions:

### Prioritize policy and funding support for GS coverage

Governments, policymakers, and healthcare systems should prioritize and allocate resources to support universal coverage of GS as a first-line test for appropriately indicated patients adhering to indications endorsed by evidence-based guidelines from expert professional societies^13^. At a minimum. this should include adequate funding and reimbursement mechanisms to enable GS for critically ill infants, pediatric patients with congenital anomalies, intellectual disability and developmental delay, and adult undiagnosed disease patients with signs and symptoms consistent with a genetic disorder. In the US, the Medical Genome Initiative supports the inclusion of GS as a covered benefit in all US state Medicaid programs, and federal guidance on appropriate integration of GS based on EPSDT guidelines. Similarly, in other high-income countries (HICs), we recommend the implementation of policies that support broad, timely, and equitable access to GS testing for all patients with suspected genetic disorders. In low- or middle-income countries (LMICs), where access to GS may be more difficult due to local resource constraints, we advocate for policies and reimbursement mechanisms that broadly support genetic testing inclusive of GS when available.

### Incorporate Health Technology Assessment (HTA) processes and cost-effectiveness assessments

In geographies that utilize health technology assessments as a component of policy decisions, we recommend the implementation of a ‘living HTA’ that incorporates ongoing assessment of both the clinical utility and cost-effectiveness of GS and genome-informed care within the local healthcare system. This approach ensures continuous evaluation and updates to the assessment methodology, aligning decisions with evolving scientific, clinical, and economic considerations in genetic testing. Additionally, we recommend the development of international evidence requirement standards and data exchange mechanisms to expedite technology reviews across geographies and disparate health systems. This approach will foster global equity in access to genomic testing and promote the timely adoption of innovative healthcare solutions across diverse healthcare systems.

### Reduce the administrative barriers

To improve access to GS, there is an urgent need to streamline pre-authorization, eliminate co-pays or other out-of-pocket expenses when insurance coverage is present, and simplify administrative procedures. Reducing administrative barriers will not only save time but also alleviate the financial burdens of patients and healthcare providers, ensuring timely access to critical genetic testing services. We note that in the US, the American Medical Association (AMA) is pursuing state-level legislation to streamline appropriate test ordering, and we anticipate similar efforts in other geographies. A comprehensive reduction in administrative burdens, is essential to further improve access to GS testing.

### Strive for equitable access from the outset

Equitable access to GS should be prioritized to ensure that all patients, regardless of their background or socioeconomic status, can benefit from a genetic diagnosis and genome-informed care. In the US, Medicaid policies, managed at the state level, must be expanded to enable access to GS in underserved populations. In other geographies, without public funding, access will be limited to patients that can afford to pay out of pocket. The clinical genetics community should work with local governments, the pharmaceutical industry, nongovernmental organizations (NGOs), and philanthropiists to support in-country capacity building and test subsidization programs.

### Continued development of evidence-based guidelines

Continued development of evidence-based guidelines that detail the indications for GS testing and genome-informed treatment are essential to widespread adoption. Guidelines should be developed in collaboration across professional societies to support awareness and utilization beyond medical genetics professionals. Gaps in the evidence should be clearly articulated to enable both academic stakeholders and private industry to develop plans to address them.

### Advocate for comprehensive care coordination

It is critical to establish mechanisms for effective care coordination throughout the diagnostic and precision medicine process to maximize patient benefit and constrain costs. This will require improved communication mechanisms across care providers and the development and implementation of infrastructure that supports timely result-sharing and coordinated follow-up for care continuity.

### Resource clinician education and training

Expanded continuing education and training programs are needed for healthcare professionals in clinical genomics and genomic medicine. This should include integrating genomics education into medical and allied health curricula and providing ongoing professional development opportunities. Clinical education and training are necessary to address equity of access issues in disadvantaged communities worldwide.

### Engage the public and raise awareness

To increase appropriate utilization of genomic testing, the public must be educated about both the strengths and limitations of these approaches, including the benefits of genome-informed treatment. Multi-stakeholder campaigns that include hospital systems, payers, professional societies, and industry, which engage prospective patients, may lead to more effective testing and improved public policy. We support public awareness campaigns that focus on historically disadvantaged and under-represented populations (e.g. indigenous communities, globally).

In summary, these actions call upon a wide range of stakeholders, including governments, healthcare systems, professional societies, educational institutions, NGOs, and industry, to collaborate to address the challenges and disparities in genetic testing access and utilization.

## Conclusion

GS has ushered in a new era in the diagnosis of genetic diseases, offering the potential for improved patient care. Now is the time for collective action to overcome challenges, implement best practices, and ensure that the benefits of GS are realized for all individuals affected by genetic diseases. Indeed, widespread and appropriate utilization of GS is critical for directing the emerging gene editing, gene therapy, and cell-based therapies for rare genetic disorders. Concerted policy, education, guideline, and care pathway efforts will drive significant advancements in precision medicine and improve health outcomes for patients with genetic conditions.

### Supplementary information

Supplement_MGI Members with Affiliation

## Data Availability

Data sharing is not applicable to this article as no datasets were generated or analyzed during the current study.
