# Supplementary material for: Advancing access to genome sequencing for rare genetic disorders: recent progress and call to action
Source: NPJ Genom Med. 2024 Mar 27;9:23. doi: 10.1038/s41525-024-00410-2 (PMC10973466; doi:10.1038/s41525-024-00410-2)
Supplement: Supplementary file 1 — Supplement_MGI Members with Affiliation [file 41525_2024_410_MOESM1_ESM.docx]

Supplement MGI members

Vaidehi Jobanputra^1,2^, Heidi L Rehm^3,4^, Christian Marshall^5^, Wei Shen^6^, Euan Ashley^7,8^, Elizabeth Spiteri^7^, Ghunwa Nakouzi^9^, Linyan Meng^10^, Pengfei Liu^10^, Stephen Kingsmore^11^, Katarzyna Ellsworth^11^, Ryan J. Taft^12^, Niall Lennon^3^, Neal Niu^6^, Michael Zody^1^, Susan Hiatt^9^, Teri Manolio^13^

^1^Molecular Diagnostics, New York Genome Center, New York, NY, USA

^2^Pathology and Cell Biology, Columbia University Medical Center, New York, NY, USA

^3^Medical and Population Genetics, Broad Institute of MIT and Harvard, Cambridge, MA, USA

^4^Center for Genomic Medicine, Massachusetts General Hospital, Boston, MA, USA

^5^Division of Genome Diagnostics, Pediatric Laboratory Medicine Department, The Hospital for Sick Children, Toronto, ON, Canada

^6^Department of Medicine and Pathology, Mayo Clinic, Rochester, MN, USA

^7^Clinical Genomics, Department of Pathology, Stanford Medicine, Palo Alto, CA, USA

^8^Stanford Center for Undiagnosed Diseases, Stanford University, Stanford, CA, USA

^9^HudsonAlpha Clinical Services Lab, LLC, HudsonAlpha Institute for Biotechnology, Birmingham, AL, USA

^10^Molecular and Human Genetics, Baylor College of Medicine, Houston, TX, USA

^11^Rady Children’s Institute for Genomic Medicine, Rady Children’s Hospital, San Diego, CA, USA

^12^Medical Genomics Research, Illumina Inc., San Diego, CA, USA

^13^**Division of Genomic Medicine,**[National Human Genome Research Institute (NHGRI)](https://www.genome.gov/)**, USA**
